# Knowledge, attitudes and practices toward childhood vaccination among guardians in Chengdu, China: a cross-sectional study

**DOI:** 10.3389/fped.2025.1511018

**Published:** 2025-07-14

**Authors:** Wen Liu, Hongxia Li, Ling Qin, Hui Wang, Kai Zhu, Taoyi Yang, Xufeng Jia, Lanqian Xu

**Affiliations:** ^1^ Department of Pediatric, The Third People’s Hospital of Chengdu, Chengdu, Sichuan, China; ^2^ Department of Ophthalmology, The Third People’s Hospital of Chengdu, Chengdu, Sichuan, China; ^3^ College of Nursing, Xinjiang Medical University, Urumqi, Xinjiang, China

**Keywords:** children's guardians, vaccines, vaccination, knowledge, attitudes, practices, influencing factors

## Abstract

**Background:**

Vaccination effectively prevents various infectious diseases and represents one of the most cost-effective health interventions. In China, childhood immunization programs have achieved remarkable success, but guardian knowledge, attitudes, and practices (KAP) regarding vaccination significantly influence immunization coverage. Understanding vaccination KAP among children's guardians in Chengdu is crucial for optimizing immunization programs.

**Aim:**

To assess knowledge, attitudes, and practices regarding childhood vaccination among guardians in Chengdu's Third People's Hospital and identify associated factors to improve vaccination service quality.

**Methods:**

A cross-sectional survey was conducted among 612 guardians of children aged 0–6 years between January and June 2023. Participants were recruited through systematic sampling during vaccination visits, telephone appointments, and household surveys. A structured questionnaire assessed vaccination knowledge (13 items), attitudes (11 items), and practices (9 items). Data were analyzed using *χ*^2^ tests and logistic regression analysis (*P* < 0.05).

**Results:**

Among 612 participants (response rate 90.1%), 97.7% were aware that newborns require vaccination within 24 hours, but knowledge gaps existed regarding vaccine classifications (56.1% aware of Category I vaccines), adverse reactions (61.8%), and specific vaccines like meningococcal (47.1%) and hepatitis A (41.5%). Most guardians (85.1%) considered vaccination necessary, and 69.9% proactively sought vaccination services. However, only 23.4% were unaffected by negative media reports. Logistic regression revealed that higher education levels (OR = 1.51, 95%CI: 1.02–2.24), higher family income (OR = 1.80, 95%CI: 1.09–2.96), and better housing conditions (OR = 1.89, 95%CI: 1.03–3.45) were associated with better vaccination knowledge.

**Conclusion:**

Guardians in Chengdu demonstrate positive attitudes toward vaccination but require improved knowledge, particularly regarding vaccine categories and safety. Targeted education programs should focus on vulnerable populations with lower education and income levels to enhance vaccination coverage and child health protection.

## Introduction

Vaccination represents one of the most effective public health interventions for preventing infectious diseases and protecting child health (1, 2). Vaccines have contributed significantly to reducing childhood morbidity and mortality worldwide, making immunization programs essential components of comprehensive healthcare systems (3).

China has implemented extensive childhood immunization programs for decades, achieving substantial improvements in child health outcomes (4). Following the promulgation of regulations on vaccine circulation and vaccination administration, various regions have actively developed immunization programs according to local conditions (5). However, vaccination policy implementation varies significantly across different local governments (6). With rapid economic development, Chengdu has experienced dramatic increases in migrant populations, with many migrant children accompanying their working parents to urban areas, creating new challenges for routine immunization management (7).

In China's National Immunization Program, vaccines are classified into two categories: Category I vaccines are mandatory and provided free of charge by the government, while Category II vaccines are optional and require out-of-pocket payment (8). This classification system significantly influences parents' vaccination decisions and knowledge needs, particularly regarding the importance and safety of Category II vaccines (9).

Parents and guardians serve as primary decision-makers for children's vaccination, and their knowledge, attitudes, and practices directly affect vaccination uptake and immunization coverage rates (10, 11). Recent studies have demonstrated positive correlations between guardian vaccination knowledge levels and successful immunization program implementation (12, 13). Furthermore, educated guardians can serve as health educators within migrant communities through “peer education” mechanisms, extending vaccination knowledge to broader populations (14).

Guardian participation in vaccination decisions proves valuable for smooth immunization program implementation and reduction of vaccine-preventable diseases (15, 16). Research from various settings has shown that comprehensive knowledge-attitude-practice (KAP) assessments provide essential insights for developing targeted health education interventions and improving vaccination services (17, 18).

However, limited research has examined vaccination KAP among guardians in major Chinese cities like Chengdu, particularly considering the unique challenges posed by mixed local and migrant populations. This study was conducted to assess vaccination knowledge, attitudes, and practices among guardians of preschool children in Chengdu, providing evidence for developing improved immunization strategies, enhancing vaccination service quality, and better controlling vaccine-preventable diseases.

## Participants and methods

### Study design and participants

This cross-sectional study was conducted at the Third People's Hospital of Chengdu, Sichuan Province, between January and June 2023. A total of 679 guardians of children aged 0–6 years were initially screened, of whom 612 (90.1%) voluntarily agreed to participate and completed the questionnaire effectively.

Guardians were defined as individuals primarily responsible for children's daily care, including parents, maternal grandparents, paternal grandparents, or other caregivers. Inclusion criteria were: guardians seeking vaccination services at the study hospital; local residence duration exceeding three months. Exclusion criteria included: children with vaccination contraindications; institutionalized children; guardians with cognitive impairments. One guardian per household was surveyed to avoid clustering effects.

### Data collection methods

Data collection employed three approaches with specific recruitment numbers:Systematic sampling during routine vaccination visits on randomly selected service days: 245 participants (40.0%).Scheduled interviews with guardians contacted via telephone appointments: 198 participants (32.4%).Community-based household surveys using random address selection: 169 participants (27.6%).

Vaccination days were randomly selected using a random number generator. Telephone contacts were systematically sampled from the hospital's immunization registry. Household surveys employed random street address selection within the hospital's catchment area.

Participants did not receive monetary compensation. On-site participants received priority service without queuing, while telephone and household survey participants received 20 yuan transportation subsidies.

### Survey instrument

The structured questionnaire was developed following extensive literature review and expert consultation (19, 20). Content validity was assessed by 13 immunization experts, achieving a Content Validity Index (CVI) of 0.87. Face validity was evaluated through cognitive interviews with 30 guardians. A pilot study with 30 participants demonstrated good internal consistency (Cronbach's *α* = 0.82) (21).

The questionnaire comprised four main sections:

#### Demographic information

Gender, household registration, occupation, education level, monthly family income, housing situation, and vaccination notification receipt.

#### Vaccination knowledge assessment (13 items)

Covering vaccination requirements, contraindications, vaccine categories, adverse reactions, and specific vaccine awareness.

#### Vaccination attitudes (11 items)

assessing perceptions of vaccination necessity, willingness to receive optional vaccines, and trust in vaccination services.

#### Vaccination practices (9 items)

Evaluating actual vaccination behaviors, appointment compliance, and health information seeking. See Supplementary Material for the complete questionnaire.

### Statistical analysis

Data were double-entered using EpiData 3.0 and analyzed with IBM SPSS 21.0 (22). Descriptive statistics included frequencies and percentages for categorical variables and means ± standard deviations for continuous variables. Knowledge scores were analyzed as continuous variables and categorized using quartile distributions for comparative analysis.

Chi-square tests examined associations between demographic characteristics and vaccination knowledge levels. Variables showing significant associations (*P* < 0.05) in univariate analysis were included in binary logistic regression models to identify independent predictors of vaccination knowledge. All statistical tests were two-tailed with significance set at *P* < 0.05.

### Ethics approval

This study was conducted according to the Declaration of Helsinki and approved by the Ethics Committee of The Third People's Hospital of Chengdu (SC-CD-34) (23). All participants provided written informed consent before participation.

## Results

### Participant characteristics

A total of 679 questionnaires were distributed and 612 were completed, yielding a response rate of 90.1%. Among participants, 311 (50.8%) were male and 301 (49.2%) were female guardians. The sample included 89 (14.5%) fathers, 121 (19.8%) mothers, and 402 (65.7%) other relatives including grandparents.

Educational levels comprised: elementary school or below 144 (23.5%), junior high school 155 (25.3%), high school/technical secondary school 181 (29.6%), and college or above 132 (21.6%). A total of 389 (63.6%) participants held urban registration, while 223 (36.4%) were non-local residents.

Monthly per capita family income distribution included: <1,000 yuan 97 (15.9%), 1,000–3,000 yuan 158 (25.8%), 3,000–5,000 yuan 176 (28.8%), and ≥5,000 yuan 181 (29.6%).

### Vaccination knowledge assessment

Guardians demonstrated highest awareness rates for newborn vaccination requirements within 24 hours (97.7%) and fever contraindications (94.1%). However, substantial knowledge gaps existed regarding Category II vaccination necessity (32.8%) and specific vaccination schedules (46.9%).

Among common childhood vaccines, guardians showed greater familiarity with hepatitis B vaccine (95.8%) and BCG vaccine (92.2%), while knowledge was limited for meningococcal vaccine (47.1%) and hepatitis A vaccine (41.5%). Regarding vaccine-preventable diseases, awareness was highest for polio (88.9%) and tuberculosis (85.3%), but lower for Japanese encephalitis (45.1%) and meningococcal disease (39.7%) (Table 1).

**Table 1:** Guardian knowledge of key vaccination topics (*n* = 612).

Knowledge area	Aware *n* (%)
Newborn vaccination within 24 h	598 (97.7)
Fever vaccination contraindication	576 (94.1)
Post-vaccination observation requirement	557 (91.0)
Vaccination prevents disease	545 (89.1)
School entry vaccination certificate	545 (89.1)
Hepatitis B vaccine awareness	586 (95.8)
BCG vaccine awareness	564 (92.2)
Oral polio vaccine awareness	555 (90.7)
Meningococcal vaccine awareness	288 (47.1)
Category II vaccination necessity	201 (32.8)

See Supplementary Table S1 for complete knowledge assessment results.

### Factors associated with vaccination knowledge

Univariate analysis revealed significant associations between vaccination knowledge and guardian education level (*χ*^2^ = 8.719, df = 3, *P* = 0.033), monthly family income (*χ*^2^ = 10.001, df = 3, *P* = 0.018), household registration status (*χ*^2^ = 10.235, df = 2, *P* = 0.006), occupation (*χ*^2^ = 10.781, df = 3, *P* = 0.013), and housing situation (*χ*^2^ = 7.027, df = 2, *P* = 0.029). No significant associations were found with child gender or vaccination notification receipt (Table 2).

**Table 2:** Factors associated with vaccination knowledge - univariate analysis.

Variable	Category	n	Lower knowledge *n* (%)	Higher knowledge *n* (%)	*χ*²	df	*P*-value
Education Level	Elementary or below	144	89 (61.8)	55 (38.2)	8.719	3	0.033
	Junior high school	155	86 (55.5)	69 (44.5)			
	High school/technical	181	83 (45.9)	98 (54.1)			
	College or above	132	58 (43.9)	74 (56.1)			
Monthly Income	<1,000 yuan	97	38 (39.2)	59 (60.8)	10.001	3	0.018
	1,000–3,000 yuan	158	65 (41.1)	93 (58.9)			
	3,000–5,000 yuan	176	87 (49.4)	89 (50.6)			
	≥5,000 yuan	181	93 (51.4)	88 (48.6)			
Housing Status	Rental housing	233	132 (56.7)	101 (43.3)	7.027	2	0.029
	Company dormitory	182	84 (46.2)	98 (53.8)			
	Owned/self-built house	197	89 (45.2)	108 (54.8)			

See Supplementary Table S2 for complete univariate analysis results.

Logistic regression analysis identified education level, family income, and housing situation as independent predictors of vaccination knowledge. Guardians with higher education levels (OR = 1.51, 95%CI: 1.02–2.24, *P* = 0.041), higher family income (OR = 1.80, 95%CI: 1.09–2.96, *P* = 0.021), and better housing conditions (OR = 1.89, 95%CI: 1.03–3.45, *P* = 0.039) demonstrated significantly better vaccination knowledge.

### Vaccination attitudes and practices

Most guardians (85.1%) considered vaccination necessary for children, while 70.6% viewed vaccination as the most cost-effective method for controlling infectious diseases. However, only 23.4% reported being unaffected by negative media coverage regarding vaccination.

Regarding vaccination practices, 69.9% of guardians proactively sought vaccination services for their children, while 41.5% were willing to pay for optional Category II vaccines. Compliance with post-vaccination observation requirements was reported by 31.9% of participants (Table 3).

**Table 3:** Guardian attitudes and practices toward vaccination.

Attitude/practice item	Positive *n* (%)	Uncertain *n* (%)	Negative *n* (%)
Vaccination is necessary	521 (85.1)	76 (12.4)	15 (2.5)
Vaccination most cost-effective	432 (70.6)	164 (26.8)	16 (2.6)
Unaffected by negative media	143 (23.4)	287 (46.9)	182 (29.7)
Proactive vaccination seeking	428 (69.9)	145 (23.7)	39 (6.4)
Willing to pay for Category II vaccines	254 (41.5)	198 (32.4)	160 (26.1)
Post-vaccination observation compliance	195 (31.9)	324 (52.9)	93 (15.2)

### Information sources and service preferences

Primary sources of vaccination information included bulletin boards/brochures (21.9%), television/radio (18.3%), relatives/fellow residents (17.5%), vaccination certificates (15.0%), and medical personnel (14.5%). Internet sources accounted for 12.8% of information seeking.

Guardians expressed greatest concern about vaccine safety (25.2%) and effectiveness (20.8%), while cost concerns were relatively lower (15.5%). Preferred appointment notification methods included vaccination certificates (23.4%) and telephone calls (20.3%).

Desired services during vaccination included enhanced physician explanations about vaccines and related knowledge (17.3%), professional consultation services (16.8%), and reduced waiting times (13.4%).

## Discussion

This cross-sectional study is the first to quantify childhood-vaccination knowledge, attitudes and practices (KAP) among guardians in a rapidly urbanising district of Chengdu that hosts a large migrant population. Three headline findings emerged. First, overall attitudes were strongly positive—more than four in five guardians endorsed the necessity of vaccination—yet substantial knowledge gaps remained, especially for Category II (self-paid) vaccines, with only one third recognising their importance. Second, socioeconomic gradients were pronounced: higher education, income and home ownership independently predicted better knowledge scores. Third, exposure to negative media reports was common and strongly associated with hesitant attitudes. Taken together, these results identify an urgent need for more nuanced, equity-focused communication strategies.

Participants in our study displayed a level of knowledge about Category II vaccines that aligns with results from an earlier investigation conducted in an inland setting, yet still trails behind the knowledge documented in a more economically developed coastal metropolis (24, 25). This gap is probably shaped by a combination of factors, including local economic conditions, the distribution of educational resources, the reach and intensity of public-health messaging, and how readily residents can access trustworthy health information. The regional discrepancy supports the notion that economic development and health-information infrastructure jointly shape vaccine awareness. Outside China, Wilson et al. reported that only 60% of French nurses recommended routine childhood immunisation (26), illustrating that professional endorsement alone does not guarantee universal parental confidence. Conversely, the 85.1% positive-attitude rate in our study exceeds the 71.0% reported in a United States HealthStyles survey during the same period (27), suggesting that guardians in Chengdu remain broadly supportive of the national immunisation programme despite media scandals.

The socioeconomic patterning we observed aligns with health-literacy theory, which posits that people with greater resources have better access to information and a higher capacity to evaluate conflicting claims (28, 29). For migrant families, structural barriers—unstable housing, time-poor employment and weaker social networks—likely limit exposure to reliable sources and magnify the impact of sensational online content. Kata has shown that anti-vaccine messages circulate rapidly on social media platforms by exploiting emotive storytelling and distrust of authorities (30). Our finding that barely a quarter of guardians remain unaffected by negative media underscores the need to inoculate parents against misinformation before crises erupt.

Based on these insights, three tiers of intervention appear feasible. **First**, strengthen point-of-care education: brief, image-rich modules delivered by vaccination nurses during observation periods can cover Category II indications, safety profiles and adverse-reaction management. Evidence from Hu et al. indicates that such micro-learning sessions significantly improve COVID-19 vaccine intent (31), and the same principle should translate to routine childhood vaccines. **Second**, leverage digital tools already embedded in daily life. Integrating a “smart reminder” function into existing WeChat health mini-programmes would allow guardians to scan the child's vaccination certificate and receive algorithm-tailored messages at key decision points, an approach supported by the favourable uptake of QR-code health passes during the pandemic. **Third**, embed vaccination messaging in migrant-friendly community venues—factory dormitories, urban villages and evening clinics—because caretakers in these settings report trusting peer advice over official brochures (32). Materials must be linguistically plain and visually guided to accommodate low functional literacy.

Economic considerations also merit attention. Although willingness to pay for Category II vaccines in our sample was only 41.5%, the figure exceeds the uptake recorded in Shanghai's migrant districts (<30%) (10). Progressive subsidy schemes—partial reimbursement for children from low-income households—could close the affordability gap while preserving consumer choice. Policymakers should pilot such schemes in high-migration wards and evaluate cost-effectiveness alongside educational interventions.

Several limitations warrant caution. The cross-sectional design precludes causal inference, and self-report may inflate desirable practices through social-desirability bias. Although we attained a 90% response rate, the single-centre setting may limit generalisability to rural Sichuan or other megacities. In addition, the KAP instrument, while demonstrating acceptable reliability (*α* = 0.82), has yet to be validated in non-Mandarin-speaking populations. Future work should (i) employ longitudinal designs to test whether tailored education improves actual Category II vaccination uptake over time, (ii) replicate our survey in multiple provinces to examine regional heterogeneity, and (iii) apply item-response theory to refine the questionnaire and establish normative cut-offs for “adequate” knowledge.

## Conclusion

Guardians in Chengdu demonstrate positive attitudes toward childhood vaccination but require enhanced knowledge, particularly regarding vaccine categories, safety profiles, and adverse reaction management. Socioeconomic factors significantly influence vaccination knowledge levels, suggesting need for targeted educational interventions among vulnerable populations. Strengthening healthcare provider communication skills and developing comprehensive, culturally appropriate educational materials could substantially improve vaccination coverage and child health outcomes in this rapidly urbanizing population.

## Data Availability

The raw data supporting the conclusions of this article will be made available by the authors, without undue reservation.
