# Supplementary material for: Knowledge, attitudes and practices toward childhood vaccination among guardians in Chengdu, China: a cross-sectional study
Source: Front Pediatr. 2025 Jul 14;13:1511018. doi: 10.3389/fped.2025.1511018 (PMC12301298; doi:10.3389/fped.2025.1511018)
Supplement: Supplementary file 1 [file Table1.docx]

**Complete Questionnaire**

**Knowledge, Attitudes and Practices Toward Childhood Vaccination Among Guardians in Chengdu, China**

**Instructions for Participants**

Dear Guardian,

Thank you for participating in this survey about childhood vaccination knowledge, attitudes, and practices. This questionnaire aims to understand your views and experiences regarding vaccination to help improve vaccination services. Your responses will remain confidential and be used for research purposes only. Please answer all questions honestly based on your knowledge and experience.

The questionnaire takes approximately 15-20 minutes to complete. There are no right or wrong answers - we are interested in your honest opinions and experiences.

**PART I: DEMOGRAPHIC INFORMATION**

**1. Child's Information**

- Child's gender: □ Male □ Female
- Child's age: _____ years _____ months
- Child's birth order: □ First child □ Second child □ Third child or more

**2. Guardian Information**

- Your relationship to the child: □ Father □ Mother □ Maternal grandmother □ Maternal grandfather □ Paternal grandmother □ Paternal grandfather □ Other: _______
- Your age: _____ years
- Your gender: □ Male □ Female

**3. Household Registration** □ Local urban resident (Chengdu city) □ Cross-district within Chengdu city □ Cross-city within Sichuan province
□ From outside Sichuan province

- How long have you lived in Chengdu? _____ years _____ months

**4. Education Level** □ Elementary school or below □ Junior high school □ High school/technical secondary school □ College/university degree □ Graduate degree or above

**5. Occupation** □ Industrial worker/blue-collar worker □ Service and sales staff □ Small trader/individual business owner □ Government employee/civil servant □ Professional/technical staff □ Student □ Unemployed/housewife □ Retired □ Other: _______

**6. Monthly Family Income (per capita)** □ Less than 1000 yuan □ 1000-3000 yuan □ 3000-5000 yuan □ 5000-8000 yuan □ More than 8000 yuan

**7. Housing Situation** □ Rented house in the community □ Company/employer dormitory □ Purchased house/apartment □ Self-built house □ Living with relatives □ Other: _______

**8. Vaccination Notification** Have you received vaccination appointment notifications from the hospital? □ Yes, regularly □ Yes, sometimes □ No, never

**PART II: VACCINATION KNOWLEDGE ASSESSMENT**

**Instructions:** Please indicate whether you know or are aware of the following vaccination-related information. Choose "Yes" if you know, "No" if you don't know, or "Uncertain" if you're not sure.

| **Knowledge Item** | **Yes** | **No** | **Uncertain** |
| --- | --- | --- | --- |
| **Basic Vaccination Requirements** |  |  |  |
| 9. Newborns need to receive vaccinations within 24 hours of birth | □ | □ | □ |
| 10. Children need to receive vaccinations up to 7 years of age | □ | □ | □ |
| 11. Vaccination certificates need to be checked when children enter school | □ | □ | □ |
| **Vaccination Safety and Procedures** |  |  |  |
| 12. Children cannot be vaccinated when they have a fever | □ | □ | □ |
| 13. There are contraindications to vaccination | □ | □ | □ |
| 14. Parents need to inform healthcare workers about the child's health status before vaccination | □ | □ | □ |
| 15. It is necessary to sign an informed consent form before vaccination | □ | □ | □ |
| 16. Children need to be observed for 15-30 minutes after vaccination | □ | □ | □ |
| **Vaccine Categories and Types** |  |  |  |
| 17. National immunization program vaccines (Category I) are free of charge | □ | □ | □ |
| 18. Category II vaccines are necessary for children's health | □ | □ | □ |
| 19. Category II vaccines are voluntary and paid by families | □ | □ | □ |
| **Specific Vaccination Knowledge** |  |  |  |
| 20. Children should not drink hot milk or breastfeed within 30 minutes after taking oral polio vaccine (OPV) sugar pills | □ | □ | □ |
| 21. Each vaccination has a specific recommended time schedule | □ | □ | □ |
| **22. Vaccination Knowledge - Specific Vaccines** |  |  |  |
| Please indicate which of the following vaccines you are familiar with: (Multiple choices allowed) |  |  |  |
| □ Hepatitis B vaccine |  |  |  |
| □ BCG vaccine (tuberculosis prevention) |  |  |  |
| □ Oral polio vaccine (OPV) sugar pills |  |  |  |
| □ Pertussis combination vaccine (DPT) |  |  |  |
| □ Measles vaccine |  |  |  |
| □ Japanese encephalitis (JE) vaccine |  |  |  |
| □ Meningococcal (MCV) vaccine |  |  |  |
| □ Hepatitis A vaccine |  |  |  |
| □ I am not familiar with any of these vaccines |  |  |  |

**23. Disease Prevention Knowledge** Please indicate which diseases you know can be prevented by vaccination: (Multiple choices allowed) □ Poliomyelitis (polio) □ Tuberculosis □ Pertussis (whooping cough) □ Measles □ Hepatitis B □ Diphtheria □ Tetanus □ Hepatitis A □ Japanese encephalitis B □ Epidemic cerebrospinal meningitis □ I don't know which diseases can be prevented by vaccines

**PART III: VACCINATION ATTITUDES**

**Instructions:** Please indicate your level of agreement with the following statements about vaccination.

| **Statement** | **Strongly Agree** | **Agree** | **Uncertain** | **Disagree** | **Strongly Disagree** |
| --- | --- | --- | --- | --- | --- |
| **24. Vaccination is necessary for children** | □ | □ | □ | □ | □ |
| **25. I am willing to give my child beneficial Category II vaccines** | □ | □ | □ | □ | □ |
| **26. I will take my child for vaccinations on time** | □ | □ | □ | □ | □ |
| **27. I want to learn about my child's vaccination status** | □ | □ | □ | □ | □ |
| **28. I am willing to attend lectures on vaccination knowledge** | □ | □ | □ | □ | □ |
| **29. Negative information reported by the media will not affect my decision about my child's vaccination** | □ | □ | □ | □ | □ |
| **30. If my child has adverse reactions after vaccination, I will not suspect the vaccine** | □ | □ | □ | □ | □ |
| **31. Vaccination is the most economical and effective means of controlling infectious diseases** | □ | □ | □ | □ | □ |
| **32. Vaccination certificates are important and need to be kept properly** | □ | □ | □ | □ | □ |
| **33. I trust the vaccination knowledge promoted by vaccination site personnel** | □ | □ | □ | □ | □ |
| **34. Although there may be a very small chance of abnormal reactions to vaccination, the benefits of vaccination far outweigh the risks** | □ | □ | □ | □ | □ |

**PART IV: VACCINATION BEHAVIORS**

**Instructions:** Please indicate how often you engage in the following vaccination-related behaviors.

| **Behavior** | **Always** | **Often** | **Sometimes** | **Rarely** | **Never** |
| --- | --- | --- | --- | --- | --- |
| **35. I take my child for Category I vaccinations according to appointments** | □ | □ | □ | □ | □ |
| **36. I pay for some optional vaccinations for my child** | □ | □ | □ | □ | □ |
| **37. I tell doctors at vaccination sites the truth about my child's health status** | □ | □ | □ | □ | □ |
| **38. I take the initiative to take my child for vaccination** | □ | □ | □ | □ | □ |
| **39. After vaccination, I complete the required observation period** | □ | □ | □ | □ | □ |
| **40. I pay attention to news and media reports about infectious disease outbreaks** | □ | □ | □ | □ | □ |
| **41. I contact the vaccination center when my address or phone number changes** | □ | □ | □ | □ | □ |
| **42. I regularly check on my child's vaccination completion status** | □ | □ | □ | □ | □ |
| **43. I read through my child's immunization schedule** | □ | □ | □ | □ | □ |

**PART V: INFORMATION SOURCES AND SERVICE PREFERENCES**

**44. What are your main sources of vaccination information?** (Choose up to 3) □ Bulletin boards/brochures at vaccination sites □ Relatives/friends/fellow residents □ Internet (websites, social media) □ Medical personnel/healthcare workers □ Television, radio, newspapers □ Vaccination certificates/records □ Community health workers □ Other: _______

**45. What vaccine-related information are you most concerned about?** (Choose up to 3) □ Safety of vaccines □ Effectiveness of vaccines
□ Precautions before and after vaccination □ Adverse reactions to vaccines □ Whether vaccines are free or need payment □ Vaccination schedule and timing □ Which vaccines are necessary vs. optional □ Other: _______

**46. What is your preferred method for receiving vaccination appointment notifications?** □ Vaccination certificate appointments □ Telephone notifications □ SMS text message notifications □ Mobile app notifications □ Notifications from medical staff during visits □ Email notifications □ Other: _______

**47. What services would you most like to receive during the vaccination process?** (Choose up to 3) □ Reduced waiting/queuing time □ More detailed explanations from doctors about vaccines and related knowledge □ Improved vaccination environment (comfort, cleanliness) □ Increased number of vaccination service days □ Reduced number of vaccine doses (combination vaccines) □ Professional physician consultation services □ Educational classes for guardians □ Clear information about optional Category II vaccines and their costs □ Better post-vaccination monitoring and follow-up □ Other: _______

**PART VI: ADDITIONAL COMMENTS**

**48. Do you have any concerns or worries about childhood vaccination?** □ Yes, please specify: _________________________________ □ No

**49. Have you ever delayed or refused vaccination for your child?** □ Yes, please specify the reason: _______________________ □ No

**50. What suggestions do you have for improving vaccination services in our hospital?**

**Thank you for your participation in this survey!**

*Your responses are valuable for improving vaccination services and protecting children's health. All information provided will be kept strictly confidential and used only for research purposes.*

**For research team use only:**

- Questionnaire ID: _______
- Date completed: _______
- Interviewer: _______
- Data entry status: _______
