# Supplementary material for: Knowledge, attitudes and practices toward childhood vaccination among guardians in Chengdu, China: a cross-sectional study
Source: Front Pediatr. 2025 Jul 14;13:1511018. doi: 10.3389/fped.2025.1511018 (PMC12301298; doi:10.3389/fped.2025.1511018)
Supplement: Supplementary file 2 [file Table2.docx]

**Supplementary Material**

**Supplementary Table S1. Complete Knowledge Assessment Results (n=612)**

| **Knowledge Item** | **Aware n (%)** | **Unaware n (%)** |
| --- | --- | --- |
| **Basic Vaccination Requirements** |  |  |
| Vaccinations are required for newborns within 24 hours of birth | 598 (97.7) | 14 (2.3) |
| Vaccinations are required for children up to 7 years of age | 236 (38.6) | 376 (61.4) |
| Vaccination certificate needs to be checked when going to school | 545 (89.1) | 67 (10.9) |
| **Vaccination Procedures and Safety** |  |  |
| Whether children can be vaccinated when they have a fever | 576 (94.1) | 36 (5.9) |
| Contraindications to vaccination | 267 (43.6) | 345 (56.4) |
| Vaccination procedures for children | 332 (54.2) | 280 (45.8) |
| Inform the children's health status before vaccination | 498 (81.4) | 114 (18.6) |
| It is necessary to sign the informed consent form | 602 (98.4) | 10 (1.6) |
| Observation in the outpatient holding area is required for 15-30min after vaccination | 557 (91.0) | 55 (9.0) |
| **Vaccine Categories and Types** |  |  |
| Types of national immunization program vaccines (Category I) and whether they are free | 343 (56.1) | 269 (43.9) |
| The necessity of Category II vaccination | 201 (32.8) | 411 (67.2) |
| Voluntary vaccination at your own expense | 446 (72.9) | 166 (27.1) |
| **Adverse Reactions and Management** |  |  |
| Adverse reactions to vaccination and how to deal with them | 378 (61.8) | 234 (38.2) |
| **Specific Vaccination Knowledge** |  |  |
| No hot milk or breastfeeding within half an hour after taking oral polio vaccine (OPV) sugar pills | 434 (70.9) | 178 (29.1) |
| The specific time of each vaccination | 287 (46.9) | 325 (53.1) |
| Vaccination can prevent corresponding diseases, which is good for children's health | 545 (89.1) | 67 (10.9) |

**Supplementary Table S2. Complete Univariate Analysis Results**

| **Variable** | **Category** | **n** | **Lower Knowledge Score n (%)** | **Higher Knowledge Score n (%)** | **χ²** | **df** | **P value** |
| --- | --- | --- | --- | --- | --- | --- | --- |
| **Child Gender** |  |  |  |  | 0.862 | 1 | 0.353 |
|  | Male | 311 | 165 (53.1) | 146 (46.9) |  |  |  |
|  | Female | 301 | 151 (50.2) | 150 (49.8) |  |  |  |
| **Guardian Education Level** |  |  |  |  | 8.719 | 3 | 0.033 |
|  | Elementary school or below | 144 | 89 (61.8) | 55 (38.2) |  |  |  |
|  | Junior high school | 155 | 86 (55.5) | 69 (44.5) |  |  |  |
|  | High school/technical secondary | 181 | 83 (45.9) | 98 (54.1) |  |  |  |
|  | College or above | 132 | 58 (43.9) | 74 (56.1) |  |  |  |
| **Guardian Household Registration** |  |  |  |  | 10.235 | 2 | 0.006 |
|  | Cross-district in the city | 196 | 104 (53.1) | 92 (46.9) |  |  |  |
|  | Cross-city in the province | 252 | 111 (44.0) | 141 (56.0) |  |  |  |
|  | Out of province | 164 | 98 (59.8) | 66 (40.2) |  |  |  |
| **Guardian Occupation** |  |  |  |  | 10.781 | 3 | 0.013 |
|  | Workers (industrial/blue-collar) | 163 | 89 (54.6) | 74 (45.4) |  |  |  |
|  | Service and sales staff | 160 | 96 (60.0) | 64 (40.0) |  |  |  |
|  | Small traders | 176 | 87 (49.4) | 89 (50.6) |  |  |  |
|  | Other | 113 | 46 (40.7) | 67 (59.3) |  |  |  |
| **Monthly Per Capita Family Income** |  |  |  |  | 10.001 | 3 | 0.018 |
|  | <1000 yuan | 97 | 38 (39.2) | 59 (60.8) |  |  |  |
|  | 1000-3000 yuan | 158 | 65 (41.1) | 93 (58.9) |  |  |  |
|  | 3000-5000 yuan | 176 | 87 (49.4) | 89 (50.6) |  |  |  |
|  | ≥5000 yuan | 181 | 93 (51.4) | 88 (48.6) |  |  |  |
| **Family Housing Situation** |  |  |  |  | 7.027 | 2 | 0.029 |
|  | Rented house in the community | 233 | 132 (56.7) | 101 (43.3) |  |  |  |
|  | Company dormitory | 182 | 84 (46.2) | 98 (53.8) |  |  |  |
|  | Purchased house/self-built house | 197 | 89 (45.2) | 108 (54.8) |  |  |  |
| **Received Vaccination Notice** |  |  |  |  | 0.056 | 1 | 0.812 |
|  | Received | 380 | 195 (51.3) | 185 (48.7) |  |  |  |
|  | Not received | 232 | 121 (52.2) | 111 (47.8) |  |  |  |
